# Recommendations for the use of early cost-effectiveness analysis to inform the health technology development process with an application to cutaneous squamous cell carcinoma

**DOI:** 10.1017/S0266462325103334

**Published:** 2025-12-12

**Authors:** Ronan Mahon, Saswata Paul Choudhury, Sekhar Kumar Dutta, Abhirup Dutta Majumdar, Bikramaditya Ghosh, Chetna Demla, Anns Thomas, Debalina Dey

**Affiliations:** 1 University of Galway, Ireland; 2 PharmaQuant International LLC, Ireland; 3 PharmaQuant Insights Pvt Ltd, India; 4 Environmental Sustainability in HTA Working Group, Health Technology Assessment international, Canada

**Keywords:** early health technology assessment, early cost-effectiveness analysis, decision modelling, manufacturer perspective, value-based pricing, value of information

## Abstract

**Objectives:**

Cost-effectiveness analyses are used to help to inform resource-allocation decision-making in healthcare systems. The manufacturers of new health technologies may choose to employ “early cost-effectiveness analysis” (eCEA) to inform the technology development process in anticipation of a value-based assessment if and when the technology is launched. We aim to provide guidance on how eCEA can effectively inform health technology development processes, presenting novel methodological approaches to address key decision-making questions.

**Methods:**

We present three core health technology development questions that eCEAs can address, as well as recommendations for deriving and presenting insights from eCEA models. A hypothetical treatment for cutaneous squamous cell carcinoma (CSCC) called “dummymab” demonstrates the analytic techniques and presentation formats.

**Results:**

We provide guidance for addressing: 1. What is a health technology’s value-based price (VBP) under a range of scenarios? 2. To what extent do different attributes of the technology contribute to its value? 3. Regarding what model parameters is further evidence most valuable? A novel net benefit approach for value driver analysis provides more reliable estimates than traditional ‘switch-on’ methods by avoiding parameter interaction effects. The manufacturer-perspective value-of-information framework enables evidence prioritization aligned with commercial decision-making while maintaining cost-effectiveness principles.

**Conclusions:**

eCEA can systematically inform technology development through value-based price estimation, value driver identification, and evidence prioritization. Implementing development decision-making based on eCEA insights can foster alignment with value-based principles of HTA-orientated decision-making systems while supporting more efficient resource allocation in technology development.

## Key points

- An early cost-effectiveness analysis can help to inform health technology development decision-making in a way that aligns with the value-based principles of CEA-orientated decision-making systems
- Methodologies are presented and demonstrated that (i) estimate value-based prices under a range of scenarios, (ii) identify the ‘value-drivers’ associated with a new health technology, and (iii) estimate the value of further evidence development from a manufacturer perspective
- The framework provides practical guidance for implementing eCEA across disease areas and technology types

## Introduction

Cost-effectiveness analysis (CEA) assesses the value of new health technologies, thereby informing resource allocation decision-making in healthcare systems (1;2). CEA is used extensively, though to different degrees, in health technology assessment (HTA) frameworks across the globe (3). A CEA involves estimating a ratio of incremental costs to incremental health benefits (typically quality-adjusted life-years (QALYs)). This incremental cost-effectiveness ratio (ICER) can then be compared against a threshold, which is typically assumed to represent the additional cost that has to be imposed on the system, such that one QALY is foregone through displacement (4). Thus, CEA can indicate whether the adoption of a new technology can be expected to add more benefit than the benefit lost through the healthcare activity displaced.

Upon regulatory approval, manufacturers typically apply for reimbursement across various healthcare jurisdictions. Reimbursement decision-making bodies may request a CEA from the manufacturer as part of a broader body of evidence required to inform an HTA process. Developing a ‘launch CEA’ may require considerable resources, but it may prove pivotal to achieving reimbursement approval in systems where cost-effectiveness is a primary criterion for decision-making (5).

A manufacturer may choose to utilize CEA methodology much earlier in the health technology development process to answer a different but related internal set of questions. An early cost-effectiveness analysis (eCEA) would typically be developed alongside phase I and phase II clinical research, where the price of the technology and the target patient population may not yet be determined. However, what will be known is a healthcare system’s willingness-to-pay for the expected QALYs gained through use of the new technology, expressed as a cost-effectiveness threshold. Therefore, an eCEA can indicate to manufacturers how cost-effectiveness might be achieved through different combinations of price, efficacy, and other factors.

From a general health economics perspective, routine use of eCEAs is to be welcomed as representing acknowledgment of and response to the value-based incentives set by healthcare systems, most conspicuously through stated cost per QALY willingness-to-pay thresholds. Effective use of eCEAs presents an opportunity for health technology development decision-making to be conducted to a greater extent through a Health Economic lens.

Though, by their nature, most eCEAs are internal to the manufacturer and usually are not published, there has been an evolving landscape of frameworks, methodologies, and applications to guide eCEA or early HTA more broadly. Mauskopf et al. advocated early integration of health economics into clinical strategies, while Sculpher et al. proposed systematic cost evaluations from the onset of clinical development (6;7). Although initially focused on pharmaceuticals, early-stage modeling extended to medical devices (8–10). Hartz and John underscored the strategic benefits of early-stage modeling in their comprehensive review (11). Miller introduced techniques such as clinical trial simulation and options pricing, while Pietzsch and Pate-Cornell and IJzerman and Steuten conceptualized early health technology assessment (HTA), focusing on medical device development and translational research respectively (12–14). Elschot et al. demonstrated the application of early-stage health economic modeling in assessing the value of risk assessment and targeted treatment, using poststroke epilepsy as an example (15). More recent research has endeavored to provide structure and a roadmap to conducting early HTA, including the role of health economic modeling. In particular, Bouttell et al. presented a framework of early HTA to provide an introduction to the space and to initiate debate, Llorian et al. presented a review of frameworks to early HTA and a roadmap to guide applications and Grutters et al. provided a critical review of published early health economic assessments (16–18). There have also been a number of recent real-world applications of eCEA, (15, 19–21), though it is worth noting that these analyses are predominantly from a system perspective. As noted by several authors, most eCEAs remain proprietary due to commercial sensitivity (22).

Although each of these contributions provides an insight into the general concepts of and the published use of early HTA, there lack of practical guidance on how to conduct eCEAs, especially from a manufacturer perspective. Building on the general roadmaps and the technical methodology outlined in recent literature, the aim of this article is to provide a practical guide as to how analysts can use eCEA to derive and present insights relating to three key health technology development questions.

## Methods

### Rationale for hypothetical case study

Publishing early assessments of specific technologies risks compromising subsequent development stages and commercial strategy. Although real-world case studies provide valuable insights (15, 19–21), a hypothetical case allows systematic illustration of all proposed methodological approaches without commercial sensitivity constraints. This approach is consistent with methodological literature in health economics where transparency of methods takes precedence over real-world application (23). We use “manufacturer” to refer to any organization developing a health technology, including pharmaceutical companies, medical device companies, and biotechnology firms.

We assess a hypothetical new treatment called *dummymab* for treating cutaneous squamous cell carcinoma (CSCC).

To ensure objectivity despite author affiliations, we implemented several measures:

- Exclusive use of published, peer-reviewed data sources (except for assumed treatment effect and price of *dummymab*)
- Strict adherence to established HTA methodological guidelines (specifically the National Institute for Health and Care Excellence (NICE) reference case)
- Transparent reporting of all assumptions and limitations
- Focus on methodological innovation rather than technology-specific conclusions

### Model background

CSCC is a type of nonmelanoma skin cancer (NMSC). The incidence of NMSC is over 100,000 cases every year in the UK, 23 percent of which is CSCC. Cemiplimab is the only approved systemic treatment for advanced CSCC in the UK. Details of previous economic evaluations in CSCC can be found in Supplementary Material A.

### Model structure and parameters

In alignment with previous economic evaluations, we developed a partitioned survival model structure to assess the cost-effectiveness of *dummymab.* Baseline patient characteristics were based on a previous study of cemiplimab (24). ECEAs are most often developed when data from comparative clinical trials are not yet available. Consequently, the assumed clinical efficacy for new technologies is typically sourced from a “target product profile” (TPP) developed by the manufacturer. The clinical efficacy for d*ummymab* was based on a hypothetical such TPP, assuming a hazard ratio of 0.50 [95% CI 0.44–0.57] for overall survival and 0.40 [95% CI 0.33–0.48] for progression-free survival compared to cemiplimab. Kaplan–Meier estimates of overall survival and progression-free survival for cemiplimab were obtained from a previous study of cemiplimab (24). Individual patient-level data were reconstructed from these Kaplan–Meier estimates based on the methodology from Guyot et al. (25). The survival curves were then extrapolated by fitting a number of standard parametric survivor functions, with the best fitting (as per Akaike Information Criterion) of these selected for use in the base case analysis (26). The hazard ratios reported in the TPP were applied to the baseline survival curve to generate survival curves for *dummymab.* Utility weights for health states, disutilities, and rates of grade 3 or 4 adverse events and rates of healthcare resource utilization were obtained from NICE Technology Appraisal 802 which assessed cemiplimab in the treatment of CSCC (27). Direct healthcare costs were included in the model using NICE reference costs. The target price of *dummymab* is assumed to be 1,723 GBP per vial, which equates to 29,963 GBP per year. The analysis assumes a UK perspective, following methods guidance from the National Institute for Health and Care Excellence (28). Details of the model inputs are provided in Supplementary Material B.

## Results

We propose three key questions pertaining to the health technology development process that eCEAs can routinely inform: (1) What is the value-based price (VBP) under a range of scenarios? (2) To what extent do different attributes of the technology contribute to its value? (3) Regarding what model parameters is further evidence required?

### What is the value-based price (VBP) under a range of scenarios?

**Figure 1.**
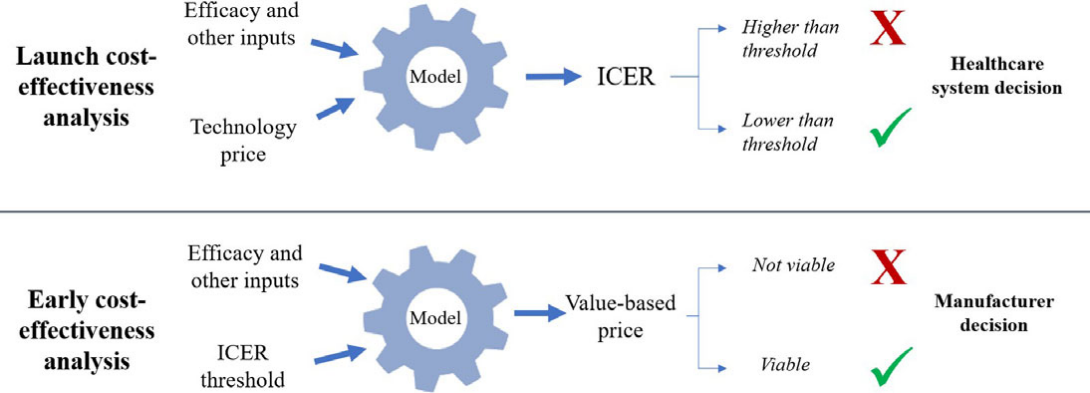
Key differences between launch and early CEA in terms of estimands and decision-making perspectives.

Value-based price (VBP) estimation shares conceptual foundations with headroom analysis but differs in application timing and decision-making context. Although headroom analysis typically occurs predevelopment to assess maximum viable development investment, VBP estimation in eCEA focuses on pricing strategies for technologies already in development (22;29). The principle, in short, is to use the eCEA to estimate the maximum price of the technology such that cost-effectiveness is achieved as per the cost-effectiveness threshold stated by the relevant decision or recommendation-making authority. This calculation involves a simple rearrangement of the decision-making formula, whereby instead of the technology price being known and the ICER being unknown, the ICER (i.e., threshold) is known and the price is unknown. As such, though the value-based principles remain the same, the decision-making onus (or opportunity) shifts to the manufacturer. Depending on the VBP calculated, the manufacturer may conclude that further development of the technology is/is not viable, or that a specific high-value population or pricing strategy ought to be pursued. These typical distinctions between early and launch CEAs are illustrated in Figure 1. Note that real-world decision-making involves additional factors beyond cost-effectiveness thresholds, including budget impact, disease severity, unmet need, and broader societal value considerations.

ECEAs can be similarly employed to isolate and estimate any currently unknown parameter value. For instance, assuming a target price enables eCEA to estimate minimum clinical efficacy satisfying both price and ICER criteria—a value-based efficacy (VBE). Such analysis may indicate the commercial viability of clinical program goals. It may be more helpful still to estimate a range of price and efficacy combinations such that the ICER criterion is satisfied. To these ends, the model needs to be programmed to find the value of the technology price parameter such that the value of the ICER output is equal to the value of the relevant cost-effectiveness threshold.

For *dummymab*, the base case VBP was estimated to be *27,574 GBP* per year, assuming a target ICER of 30,000 GBP per QALY. The base case analysis settings and assumptions can be found in Supplementary Material D. Total QALYs, total costs, and disaggregated costs by treatment are presented in Table 1. Note that the ratio of total incremental costs to total incremental QALYs is equal to 30,000 GBP, that is, the target ICER.

**Table 1.** Total QALYs, total costs, and disaggregated costs of the base case analysis

	Dummymab	Cemiplimab	Difference
Total QALYs	4.44	3.36	1.09
Drug costs (GBP)	46,819	42,403	4,416
Drug admin costs (GBP)	6,131	4,733	1,398
Medicine mgmt. costs (GBP)	99,119	70,794	28,325
Terminal care costs (GBP)	3,037	4,514	–1,477
Adverse event costs (GBP)	310	310	0
Total costs (GBP)	206,035	131,596	32,661

**Figure 2.**
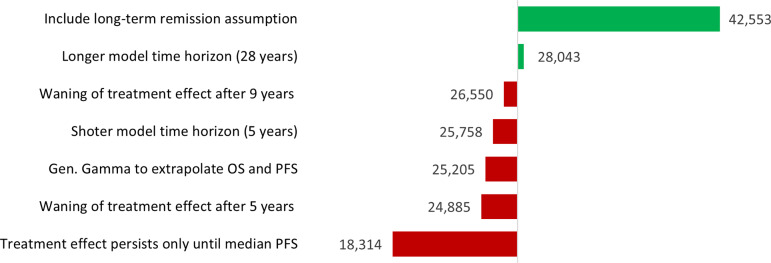
Scenario analysis results in terms of VBP per annum per patient (GBP).

There are likely to be several other scenarios that manufacturers would want to explore before making development and pricing decisions. For *dummymab*, the results of a scenario analysis are illustrated in Figure 2. Based on this analysis, the manufacturer may wish to seek to better understand the role of long-term remission in CSCC, as the inclusion of this assumption dramatically increases the VBP of a new treatment. A two-way sensitivity analysis can be found in Supplementary Material C, where VBP results are presented with respect to different combinations of OS and PFS HRs.

### To what extent do different attributes of the technology contribute to its value?

The “value” associated with a new technology may be driven by numerous factors. Several value frameworks have been proposed for appraising new health technologies (30–34). The majority of HTA frameworks in use today are variants on a basic “cost per QALY” framework. The present analysis assumes a cost per QALY framework, specifically the reference case presented by NICE in the UK (28).

The value that *dummymab* represents from a healthcare system perspective will thus relate to one or more of the components of costs and QALYs, that is, cost savings, improvement in overall survival and improvement in health-related quality of life (HRQoL). The attributes of a new technology that are expected to contribute to its value are typically outlined in a TPP (35). An eCEA can shed light on which attributes will add value from a healthcare system perspective and to what extent.

There are two methods to estimate value drivers in an eCEA. With either method, the results can be helpfully illustrated as a “waterfall” chart. The first method (“switch on”) involves setting the parameter values of the new technology to be equal to the parameter values of the comparator technology and then one-by-one “switching on” each parameter to the new technology value and in each case recording the effect on the VBP, eventually reaching the full estimate of the VBP. The “switch on” method allows the modeler to break the VBP estimate down into as many specific parameters as necessary and the output, given in terms of VBP, is easily interpretable. In particular, the impact of the anticipated sources of clinical benefit as described in a TPP (e.g., OS benefit and PFS benefit) can be investigated using this method. However, because most CEA models are dynamic (the model parameters interact) and are nonlinear (they interact multiplicatively), the estimates from the switch-on method will vary depending on the order in which the parameter values are switched on and thus may be inherently unreliable. See Supplementary Material F for an illustration of this problem using the *dummymab* example. The “switch on” method also expresses value drivers in terms of input parameters, but not in terms of the posterior health economic benefit, that is, survival, HRQoL, and cost savings.

The second method available (“net benefit”) involves calculating disaggregated QALYs and disaggregated costs in terms of incremental net benefit. Incremental net monetary benefit (INMB) is calculated as per Equation 1 where *Q*_1_ and *Q*_2_ are the estimated QALYs for technology 1 and technology 2, respectively, *C*_1_ and *C*_2_ are the estimated costs for technology 1 and technology 2, respectively and λ is the cost-effectiveness threshold.

**Equation 1**. Incremental net monetary benefit equation.
(1)

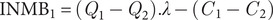



The expected QALYs gained via the new technology can be disaggregated into improved survival and improved HRQoL as follows: QALYs gained associated with improved survival can be estimated as the product of the additional life-years gained and the HRQoL of the most progressed health state (i.e., an estimate of the QALYs gained if the new technology *only* extended life), as per Equation 2. QALYs gained associated with improved HRQoL can be estimated as the remainder of the total QALYs gained as per Equation 3.

**Equation 2**. Calculation of incremental net monetary benefit associated with improved survival, where LY is life-years and HRQoL_MPS_ is the HRQoL associated with the most progressed health state
(2)

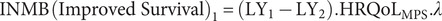


**Equation 3**. Calculation of incremental net monetary benefit associated with improved HRQoL.
(3)

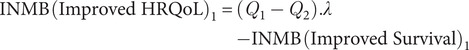



The cost-related value drivers can be estimated as per the disaggregated cost results in Table 1. The key advantage of the net benefit approach is that it can produce reliable estimates of proportional value contribution associated with each health economic output of the analysis. This method requires explicit inclusion of the target price.

**Figure 3.**
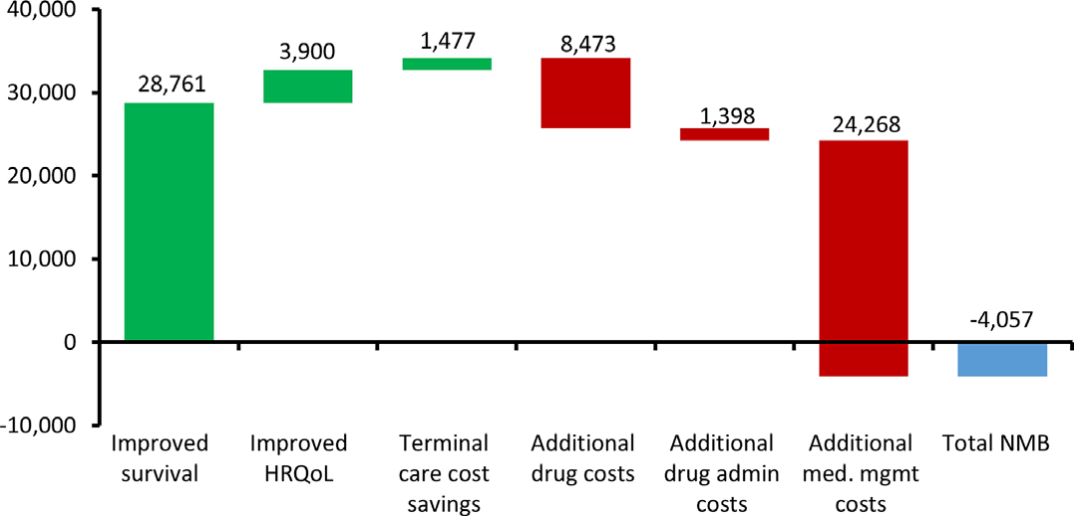
Value drivers of dummymab using the ‘net benefit’ method expressed as GBP per patient. The green bars indicate where an attribute is adding to the value of dummymab, whereas the red bars indicate where an attribute is reducing the value of dummymab.

For *dummymab*, the value driver assessment using the net benefit method is illustrated in Figure 3.

### Regarding what model parameters is further evidence required?

The question of optimal further evidence development is a function of a number of factors: the uncertainty around input parameters, the sensitivity of cost-effectiveness results to input parameters and the cost and feasibility of developing evidence. Each of these factors can be analyzed in isolation, by, respectively, characterization of input parameter uncertainty (see Supplementary Material B), one-way sensitivity analysis (see Supplementary Material E), and study costing. However, it is the combination of these factors that would ideally inform evidence development decision-making. Value-of-information (VoI) analysis is a methodology developed to inform the assessment of the need for evidence and the consequences of an uncertain decision from a healthcare system perspective (36). Two central calculations in this methodology are: the expected value of perfect information (EVPI) and the expected value of perfect information about a parameter (EVPPI). The cost of the relevant evidence being less than EVPI or EVPPI is a necessary but not sufficient condition for the worthwhile pursuit of such evidence.

VoI analyses are generally not required at the point of launch, providing manufacturers little incentive to incorporate VoI into their presentation of evidence. Some research has been conducted into how uncertainty and risk can be incorporated into technology pricing decisions (37). Other research has proposed a VoI framework from the manufacturer’s perspective based on a company’s return on investment (38). What we propose complements this previous research by outlining a version of VoI methodology that explicitly takes the perspective of the manufacturer and can easily be implemented based on an eCEA. The output can be used to routinely inform evidence development decisions in a way that aligns with the cost-effectiveness-based decision-making systems of HTA countries.

The methodology proposed here employs the following simplifying assumptions: (i) the target price of the new technology is fixed, that is, this will be the price at launch, (ii) reimbursement success depends solely on demonstrated cost-effectiveness, (iii) the costs of launch are assumed to be zero, (iv) manufacturing costs are assumed to be zero, (v) the manufacturer makes a binary launch decision based on expected cost-effectiveness.

Under this framework, manufacturer and healthcare system perspectives align regarding decision uncertainty (the probability that the target price is less than or equal to the value-based price equals the probability that the technology is cost-effective at the submitted price), though real-world adoption decisions often involve considerations beyond cost-effectiveness. Importantly, payoffs associated with decision outcomes differ between perspectives. A summary comparison between the traditional healthcare system VOI and manufacturer-focused VOI is presented in Supplementary Material G.

To estimate EVPI_M_, the expected payoffs to the manufacturer with perfect information and with current information must be calculated.

**Expected manufacturer payoff with perfect information** is the probability that the new technology is cost-effective multiplied by the lifetime costs (to the healthcare system) of the technology. In other words, the proportion of realizations of perfect evidence whereby the technology is cost-effective at the target price and therefore reimbursed.

**Expected manufacturer payoff with current information** is zero in the case that current evidence suggests cost-ineffectiveness (as it is assumed the technology definitely will not be launched) or is equal to the lifetime costs of the technology in the case that current evidence suggests cost-effectiveness (as it is assumed the technology would be launched and successfully reimbursed based on current evidence and thus there would be no value in further evidence acquisition).

The probabilities required can be estimated via a standard probabilistic sensitivity analysis (PSA). A formulation of EVPPI_M_ can be developed by isolating a specific parameter of interest *φ* and allowing only this parameter to be probabilistic in the PSA. The formulations of EVPI_M_ and EVPPI_M_ are presented in Equations 4 and 5. To obtain population EVPI_M_ and EVPPI_M_ (against which the cost of evidence acquisition can be compared), the EVPI_M_ and EVPPI_M_ estimates are multiplied by the (discounted) annual incident population for however many years the decision problem is expected to remain relevant.

**Equation 4**. EVPI from the manufacturer’s perspective, where TP = target price, VBP = value-based price, C = lifetime costs of technology per patient. *This term = C in the case where the technology is expected to be cost-effective based on current evidence.
(4)

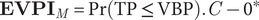


**Equation 5**. EVPPI from the manufacturer perspective where φ is the uncertain parameter of interest.
(5)

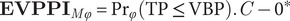



**Figure 4.**
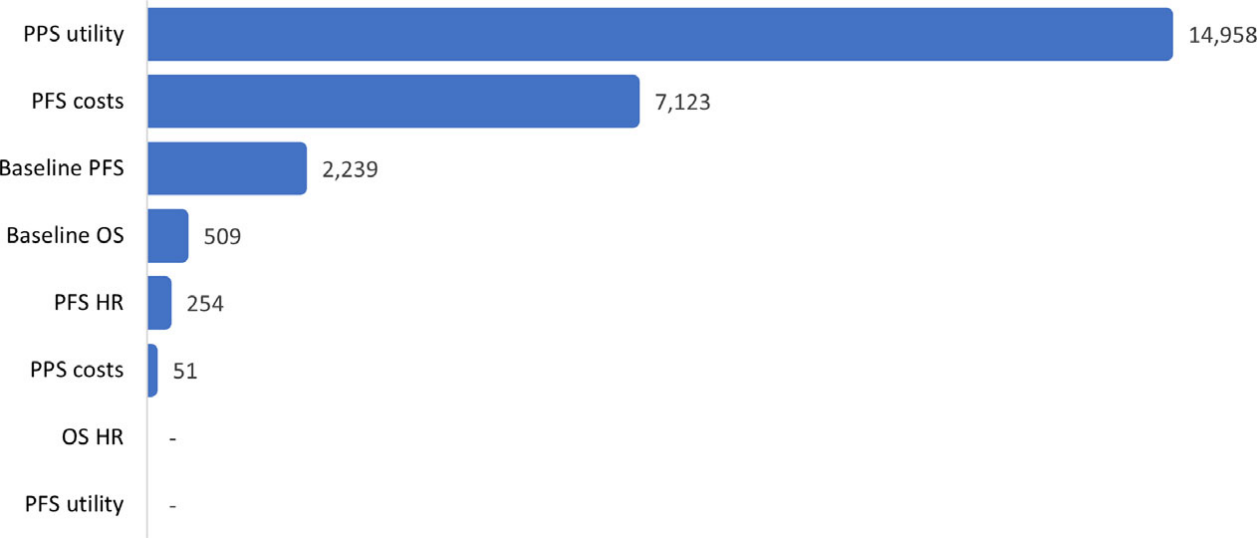
Dummymab EVPPI from the manufacturer perspective (GBP/patient).

For *dummymab*, EVPI/patient was estimated to be 24,013 GBP, that is, a probability of being cost-effective of 0.472 multiplied by the lifetime treatment costs of 50,876 GBP. The figure of 0.472 can be interpreted as: obtaining perfect information in this CEA is 47.2 percent likely to reveal a situation in which *dummymab* is shown to be cost-effective at the target price and therefore would be successfully launched. Before considering EVPPI_M_, note that since the target price (29,963 GBP) is greater than the value-based price (27,574 GBP), it is assumed that the manufacturer will not launch *dummymab* based on current evidence. Therefore, only evidence that has the potential to increase the value-based price of *dummymab* is valuable. For example, because of an age-related upper bound on utility values used in the model, more evidence regarding PFS utility only has the scope to make dummymab even less cost-effective and therefore will not change the manufacturer’s current launch decision. EVPPI, per several parameters of interest, is present in Figure 4.

## Discussion

### Generalizability across disease areas

The three-question framework presented is applicable across therapeutic areas, though some disease-specific considerations may influence implementation:

Oncology: High willingness-to-pay thresholds and end-of-life premiums may increase VBPs; survival extrapolation is often a significant source of uncertainty in the context of VoI analysis; value drivers often emphasize overall survival benefits.

Rare Diseases: Sparse data may significantly increase uncertainty and may affect the reliability of eCEA estimates; and the small patient populations also affect population-level VoI calculations; orphan drug designations may modify cost-effectiveness thresholds.

Chronic Conditions: Long-term treatment effects are an important source of effectiveness and uncertainty; value drivers often balance efficacy gains against treatment burden; quality of life improvements may outweigh survival extensions.

Medical Devices: Both upfront capital costs and recurring consumable costs affect VBP calculations; learning curves influence effectiveness parameters; value drivers may include procedural efficiency gains and healthcare resource savings.

### Other insights that eCEA can provide

Beyond the three technology development questions addressed in this analysis, eCEA can provide insights regarding other aspects of technology development or a global HEOR program. Development of an early model provides the opportunity to assess the strengths and weaknesses of the current modelling approach and to identify what further model development may be required in order to produce a robust CEA model at the time of launch.

Expanding on the VBP analysis outlined in the sections above, an eCEA can also be employed to identify scenarios such that the technology target price is economically justifiable, that is, approximately equal to the base case VBP. Such a scenario may comprise some combination of: price, patient population, treatment regimen, certain model assumptions and structures, and so forth. These insights can inform the direction of technology development and evidence generation by focusing on uses of the technology which represent most value to healthcare systems.

### Other market access considerations

The analytic framework outlined in this paper implies a single, HTA-orientated market. In reality, there are several healthcare markets that a technology manufacturer would need to consider, each with different cost per health outcome thresholds, volume potentials, and so on. Moreover, as much as a health economist may wish to view the technology development process through a lens of health economics, the realities of the global market for health technologies are such that there are several other (nonhealth economic) factors that require consideration to maximize market access success for a new health technology. The largest healthcare markets remain relatively non-HTA orientated, namely: the USA, Germany and China, and Japan. Technology pricing in particular will inevitably be largely based on commercial analysis of these markets. In general, there are many factors that inform technology pricing and development decisions, including the competitive landscape, reimbursement environments, and company commercial priorities.

The assumption that the current target price of a new technology is fixed is perhaps a strong assumption, but may be true in circumstances where there is a high “cost of goods” or there is some other strategic reason for little downward flexibility on price.

### Extension of value-of-information framework

The manufacturer’s perspective value-of-information framework outlined in this paper could be extended by relaxing some or all of the simplifying assumptions made. An extended framework may include the following conditions: there are costs associated with technology launch; cost-effectiveness at an early stage does not guarantee reimbursement success; the target price is not fixed but may evolve based on emerging evidence; the maximand from a manufacturer’s perspective is profit (that is taking manufacturing and other costs into account) rather than just cumulative sales. It is intended to develop such an extended framework as part of future research.

### Policy implications and implementation considerations

Widespread eCEA adoption has several implications for health technology ecosystems: For HTA Agencies, earlier engagement with manufacturers using eCEA could facilitate more productive dialogue about evidence requirements. For Regulators, eCEA integration into development pipelines could promote technologies better aligned with healthcare system priorities, potentially streamlining approval and reimbursement processes. For Healthcare Systems, the routine use of eCEA may result in more technologies with a high likelihood of cost-effectiveness, potentially improving the efficiency of healthcare resource allocation.

Successful eCEA implementation requires access to appropriate modeling expertise; availability of early clinical and economic data; organizational willingness to make development decisions based on economic evidence, and mechanisms for HTA agency early scientific advice.

### Limitations

Several limitations warrant acknowledgment: (i) The manufacturer’s VOI framework makes simplifying assumptions that may not reflect all real-world complexities. (ii) ECEAs rely on limited early-stage data, introducing substantial uncertainty. (iii) Although the framework applies across disease areas, specific implementations require adaptation to therapeutic area characteristics and healthcare system contexts. (iv) The methodological approaches presented require validation through real-world applications and comparison with actual development outcomes.

### Future research

Future research should address: (i) real-world case studies demonstrating eCEA impact on development decision-making; (ii) extensions of the manufacturer VOI framework incorporating additional complexities; (iii) integration of broader value elements beyond standard cost-per-QALY frameworks; (iv) development of software tools facilitating eCEA implementation, and (v) evaluation of eCEA impact on technology development efficiency and market access success.

## Conclusion

Early CEA can systematically inform the technology pricing and development process in three principal ways: (i) estimation of a value-based price, (ii) identification of the ‘drivers’ of value, and (iii) estimation of the value of further evidence acquisition. The methods outlined in this paper are intended to support routine use of eCEAs. Basing development decision-making on the insights from eCEAs can foster alignment with the value-based principles of HTA-orientated decision-making systems.

## Supporting information

10.1017/S0266462325103334.sm001 Mahon et al. supplementary material Mahon et al. supplementary material

## Supplementary material

The supplementary material for this article can be found at http://doi.org/10.1017/S0266462325103334.
