# Supplementary material for: Recommendations for the use of early cost-effectiveness analysis to inform the health technology development process with an application to cutaneous squamous cell carcinoma
Source: Int J Technol Assess Health Care. 2025 Dec 12;42(1):e7. doi: 10.1017/S0266462325103334 (PMC12835831; doi:10.1017/S0266462325103334)
Supplement: Mahon et al. supplementary material [file S0266462325103334sup001.docx]

# Supplementary materials

## Supplementary Material A: Previously Published Economic Evaluations in Advanced CSCC

| Author | Konidaris et al.2021[34] | Paul et al.2021[35] | NICE HTA (TA802)[22] |
| --- | --- | --- | --- |
| Title | Assessing the Value of Cemiplimab for Adults With Advanced Cutaneous Squamous Cell Carcinoma: A Cost-Effectiveness Analysis | Cost-effectiveness analysis of cemiplimab vs pembrolizumab for treatment of advanced cutaneous squamous cell carcinoma | Cemiplimab for treating advanced cutaneous squamous cell carcinoma |
| Intervention | Cemiplimab | Cemiplimab | Cemiplimab |
| Comparators | Historic SoC (weighted mixture of cetuximab, erlotinib, and gefitinib) | Pembrolizumab | BSC (no active treatment)  Chemotherapy (such as  platinum-based chemotherapy  and fluorouracil) |
| Population | People with metastatic cutaneous squamous cell carcinoma or locally advanced cutaneous squamous cell carcinoma who are not candidates for curative surgery or curative radiotherapy | People with metastatic cutaneous squamous cell carcinoma or locally advanced cutaneous  squamous cell carcinoma who are not candidates for curative surgery or curative radiotherapy | People with metastatic cutaneous squamous cell carcinoma or locally advanced cutaneous  squamous cell carcinoma who are not candidates for curative surgery or curative radiotherapy |
| Location/setting | US | US | UK |
| Analytical approach | Partitioned survival model | Partitioned survival model | Partitioned survival model |
| Time horizon | Lifetime (30 years) | Lifetime (30 years) | Lifetime (30 years) |
| Study perspective | US payer perspective | US perspective | NHS England perspective |
| Clinical outcomes | PFS and OS outcomes | PFS and OS outcomes | PFS and OS outcomes |
| Effectiveness data | For the cemiplimab arm - individual patient data from a phase 2 single arm trial (NCT27060498).  For SOC, analysis was based on a pooled analysis of single-arm clinical trials and retrospective studies evaluating chemotherapy and epidermal growth factor receptor inhibitors (cetuximab, erlotinib, and gefitinib) identified via a systematic literature review | For the cemiplimab arm - EMPOWER-CSCC-1, 2019 data cutoff  For the pembrolizumab arm – KEYNOTE-629,scenario analysis evaluates pooled pembrolizumab studies | For the cemiplimab arm - from an integrated analysis of data from the Phase I and Phase II EMPOWER-CSCC 1 clinical trials  For BSC ans chemotherapy arm – published literature |
| Cost data | - US list price for cemiplimab - AnalySource database (wholesale acquisition) - Centers for Medicare and Medicaid Services 2020 - HCUP - Published literature | - Published literature, - ProspectoRx. February 2021, US drug prices, - Centers for Medicare and Medicaid Services 2020 - HCUP - United States Bureau of Labor Statistics. Consumer Price Index. 2020 | - electronic Market information tool (eMit) - NHS Reference costs 2016/17. - PSSRU 2017 - Previously published NICE technical appraisals - The NICE DSU report |
| Utility and valuation | - Phase 2 cemiplimab trial (EORTC-QLQC30 data mapped to EQ-5D-3L) - Published literature | - Published literature, - Phase 2 cemiplimab trial (R2810-ONC-1540) | - Phase 2 cemiplimab trial (EORTC-QLQC30 data mapped to EQ-5D-3L) |
| Analysis of uncertainty | One-way sensitivity analysis, probabilistic sensitivity analysis | One-way sensitivity analysis, probabilistic sensitivity analysis | One-way sensitivity analysis, probabilistic sensitivity analysis |
| Discount rate | 3% | 3% | 3.5% |
| Results | Cemiplimab accrued an additional $99,447/QALY vs SOC, with higher total costs ($597,159 vs $225,051) due to increased drug acquisition and disease management. It added 4.65 LYs and 3.74 QALYs compared to SOC. cemiplimab had a 90% probability of being cost-effective when compared with historical SOC at a WTP threshold of $150,000/QALY | Patients treated with cemiplimab accrued 3.44 LYs and 2.78 QALYs when compared to pembrolizumab, with higher costs ($683,061 vs $320,817), resulting in an ICER of $130,329/QALY. Cemiplimab had a 71% probability of being cost-effective when compared with pembrolizumab at a WTP threshold of $150,000/QALY | Both base-case ICERs (presented by manufacturer and ERG) for cemiplimab vs. best supportive care were £30,952/QALY. In all scenario analysis ICERs were below £50,000/QALY. |
| Keys: best supportive care; DSU, Decision Support Unit; eMit, electronic Market information tool; ERG, evidence review group; HCUP, Agency for Healthcare Research and Quality. Healthcare cost and utilization project; ICER, incremental cost-effectiveness ratio; NICE, National Institute for Health and Care Institute; NHS, National Health System; OS, overall survival; PFS, progression free survival; QALY, quality-adjusted life years; SoC, standard of care; WTP, willingness to pay | | | |

## Supplementary Material B: Illustration of model structure and details of model parameters


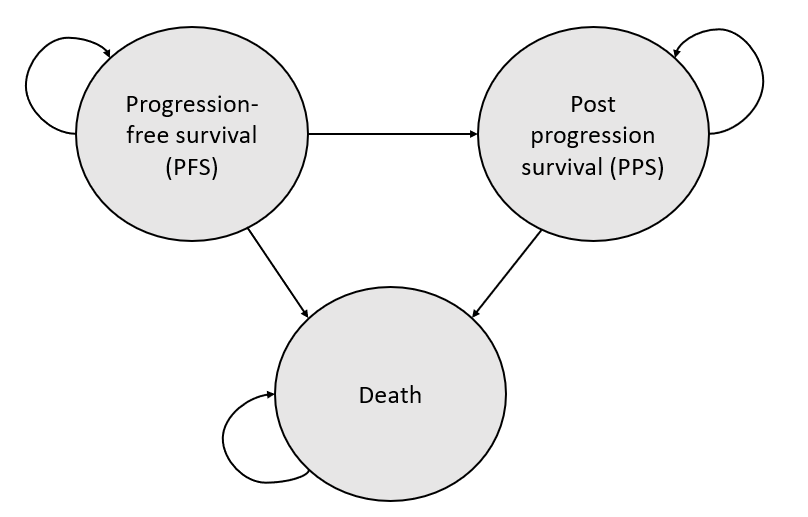


| **S.No.** | **Parameter** | **Base value** | **SE** | **α** | **β** | **Lower bound** | **Upper bound** | **Distribution** | **Source/assumptions** |
| --- | --- | --- | --- | --- | --- | --- | --- | --- | --- |
| **1** | **General model settings** |  |  |  |  |  |  |  |  |
| 2 | Model duration (years) | 10.00 |  |  |  | 2.00 | 10.00 | N/A | Assumption |
| 3 | Cycle length (days) | 30.40 |  |  |  | - | - | Fixed | Assumption |
| 4 | Discount rate for costs | 3.50% |  |  |  | 0% | 6% | N/A | Gold et al.,1996 |
| 5 | Discount rate for QALYs | 3.50% |  |  |  | 0% | 6% | N/A | Gold et al.,1996 |
| 6 | Discount rate for LYs | 0.00% |  |  |  | 0% | 6% | N/A | Gold et al.,1996 |
| 7 | Treatment effect waning | None |  |  |  | 5.00 | 10.00 |  |  |
| **8** | **Patient Characteristics** |  |  |  |  |  |  |  |  |
| 9 | Patient age | 72.00 | 7.20 |  |  | 57.89 | 86.11 | Normal | Rischin D, et al 2021 |
| 10 | Proportion of male patients | 83.40% | 8.34% | 15.77 | 3.14 | 64.16% | 96.04% | Beta | Rischin D, et al 2021 |
| 11 | Month at which patients are assumed to move to LTR | 30.00 | 3.00 |  |  | 24.12 | 35.88 | Normal | Assumption |
| 12 | Risk ratio of long-term mortality risk vs general all-cause mortality risk | 2.00 | 0.07 |  |  | 1.75 | 2.29 | Log-Normal | Assumption |
| **13** | **Treatment efficacy assumption** |  |  |  |  |  |  |  |  |
| 14 | PFS HR: Dummymab vs. Cemiplimab | 0.40 | 0.09 |  |  | 0.33 | 0.48 | Log-Normal | Based on the assumed TPP for dummymab |
| 15 | PFS HR: Cemiplimab (reference) | 1.00 | - |  |  | - | - | - |  |
| 16 | OS HR: Dummymab vs. Cemiplimab | 0.50 | 0.07 |  |  | 0.44 | 0.57 | Log-Normal | Based on the assumed TPP for dummymab |
| 17 | OS HR: Cemiplimab (reference) | 1.00 | - |  |  | - | - | - |  |
| **18** | **Fitted KM curves inputs** |  |  |  |  |  |  |  |  |
| 19 | PFS – Gen. Gamma distribution – mu | 1.05 | 0.10 |  |  | 0.84 | 1.25 | Normal | Rischin D, et al 2021 integrated analysis |
| 20 | PFS – Gen. Gamma distribution – sigma | -0.28 | 0.03 |  |  | -0.33 | -0.22 | Normal | Rischin D, et al 2021 integrated analysis |
| 21 | PFS – Gen. Gamma distribution – Q | -4.28 | 0.43 |  |  | -5.11 | -3.44 | Normal | Rischin D, et al 2021 integrated analysis |
| 22 | PFS – Exponential distribution – log(scale) | -3.37 | 0.34 |  |  | -4.02 | -2.71 | Normal | Rischin D, et al 2021 integrated analysis |
| 23 | PFS – Weibull distribution – log(scale) | 3.43 | 0.34 |  |  | 2.76 | 4.10 | Normal | Rischin D, et al 2021 integrated analysis |
| 24 | PFS - Weibull distribution - log(shape) | -0.13 | 0.01 |  |  | -0.16 | -0.11 | Normal | Rischin D, et al 2021 integrated analysis |
| 25 | PFS - Log−normal distribution - log(scale) | 2.92 | 0.29 |  |  | 2.35 | 3.49 | Normal | Rischin D, et al 2021 integrated analysis |
| 26 | PFS - Log−normal distribution - log(shape) | 0.41 | 0.04 |  |  | 0.33 | 0.49 | Normal | Rischin D, et al 2021 integrated analysis |
| 27 | PFS - Log−logistic distribution - log(scale) | 2.91 | 0.29 |  |  | 2.34 | 3.48 | Normal | Rischin D, et al 2021 integrated analysis |
| 28 | PFS - Log−logistic distribution - log(shape) | 0.08 | 0.01 |  |  | 0.07 | 0.10 | Normal | Rischin D, et al 2021 integrated analysis |
| 29 | PFS - Gompertz distribution - scale | -2.89 | 0.29 |  |  | -3.45 | -2.32 | Normal | Rischin D, et al 2021 integrated analysis |
| 30 | PFS - Gompertz distribution - shape | -0.05 | 0.01 |  |  | -0.06 | -0.04 | Normal | Rischin D, et al 2021 integrated analysis |
| 31 | OS - Gen. Gamma distribution - mu | 2.98 | 0.30 |  |  | 2.40 | 3.57 | Normal | Rischin D, et al 2021 integrated analysis |
| 32 | OS - Gen. Gamma distribution - sigma | 0.79 | 0.08 |  |  | 0.63 | 0.94 | Normal | Rischin D, et al 2021 integrated analysis |
| 33 | OS - Gen. Gamma distribution - Q | -2.27 | 0.23 |  |  | -2.71 | -1.82 | Normal | Rischin D, et al 2021 integrated analysis |
| 34 | OS - Exponential distribution - log(scale) | -4.41 | 0.44 |  |  | -5.28 | -3.55 | Normal | Rischin D, et al 2021 integrated analysis |
| 35 | OS - Weibull distribution - log(scale) | 4.45 | 0.45 |  |  | 3.58 | 5.33 | Normal | Rischin D, et al 2021 integrated analysis |
| 36 | OS - Weibull distribution - log(shape) | -0.03 | 0.003 |  |  | -0.04 | -0.03 | Normal | Rischin D, et al 2021 integrated analysis |
| 37 | OS - Log−normal distribution - log(scale) | 4.30 | 0.43 |  |  | 3.46 | 5.15 | Normal | Rischin D, et al 2021 integrated analysis |
| 38 | OS - Log−normal distribution - log(shape) | 0.55 | 0.06 |  |  | 0.44 | 0.66 | Normal | Rischin D, et al 2021 Integrated analysis |
| 39 | OS - Log−logistic distribution - log(scale) | 4.18 | 0.42 |  |  | 3.36 | 5.00 | Normal | Rischin D, et al 2021 Integrated analysis |
| 40 | OS - Log−logistic distribution - log(shape) | 0.06 | 0.01 |  |  | 0.04 | 0.07 | Normal | Rischin D, et al 2021 Integrated analysis |
| 41 | OS - Gompertz distribution - scale | -4.11 | 0.41 |  |  | -4.92 | -3.31 | Normal | Rischin D, et al 2021 integrated analysis |
| 42 | OS - Gompertz distribution - shape | -0.03 | 0.00 |  |  | -0.03 | -0.02 | Normal | Rischin D, et al 2021 Integrated analysis |
| **43** | **Costs** |  |  |  |  |  |  |  |  |
| 44 | Administration costs- Dummymab | £300.51 | £30.05 | 100.00 | 3.01 | £244.51 | £362.21 | Gamma | Assumed equal to cemiplimab |
| 45 | Administration costs- Cemiplimab | £300.51 | £30.05 | 100.00 | 3.01 | £244.51 | £362.21 | Gamma | NICE TA802 FAD |
| 46 | PFS costs (per model cycle) | £1,358.40 | £135.84 | 100.00 | 13.58 | £1,105.25 | £1,637.27 | Gamma | TA592 |
| 47 | PPS costs (per model cycle) | £879.33 | £87.93 | 100.00 | 8.79 | £715.46 | £1,059.85 | Gamma | TA592 |
| 48 | Terminal Care Costs | £8,125.05 | £812.50 | 100.00 | 81.25 | £6,610.86 | £9,793.04 | Gamma | TA592 |
| **49** | **Utilities** |  |  |  |  |  |  |  |  |
| 50 | PFS utility | 0.793 | 0.14 | 6.14 | 1.60 | 0.47 | 0.980 | Beta | TA592 |
| 51 | PPS utility | 0.701 | 0.18 | 4.10 | 1.75 | 0.31 | 0.963 | Beta | TA592 |
| 52 | Hypercalcaemia utility decrement | 0.007 | 0.02 | 4382.44 | 0.00 | -0.02 | 0.036 | Location Shifted Gamma | TA592 |
| 53 | Hypokalaemia utility decrement | 0.090 | 0.02 | 3680.44 | 0.00 | 0.06 | 0.119 | Location Shifted Gamma | TA592 |
| 54 | Skin infection utility decrement | 0.010 | 0.01 | 39204.00 | 0.00 | 0.00 | 0.020 | Location Shifted Gamma | TA592 |
| 55 | Severe skin infection utility decrement | 0.010 | 0.01 | 39204.00 | 0.00 | 0.00 | 0.020 | Location Shifted Gamma | TA592 |
| 56 | Colitis utility decrement | 0.038 | 0.00 | 64088.92 | 0.00 | 0.03 | 0.045 | Location Shifted Gamma | TA592 |
| 57 | Dummymab-specific net utility decrement | 0.007 | 0.00 | 1752152.53 | 0.00 | 0.01 | 0.009 | Location Shifted Gamma | TA592 |
| 58 | Cemiplimab-specific net utility decrement | 0.007 | 0.00 | 1752152.53 | 0.00 | 0.01 | 0.009 | Location Shifted Gamma | TA592 |
| 59 | Hepatitis utility decrement | 0.038 | 0.00 | 64088.92 | 0.00 | 0.045 | 0.352 | Location Shifted Gamma | TA592 |
| **60** | **Adverse events** |  |  |  |  |  |  |  |  |
| 61 | AE cost - Dummymab | £310.30 | £31.03 | 100.00 | 3.10 | £252.47 | £374.00 | Gamma | Assumed equal to cemiplimab |
| 62 | AE cost - cemiplimab | £310.30 | £31.03 | 100.00 | 3.10 | £252.47 | £374.00 | Gamma | TA592 |

## Supplementary Material C: VBPs for given combinations of OS and PFS hazard ratios such that the ICER = 30,000 GBP

|  |  | **PFS HR** | | | | | | | | | | |
| --- | --- | --- | --- | --- | --- | --- | --- | --- | --- | --- | --- | --- |
|  |  | **0** | **0.1** | **0.2** | **0.3** | **0.4** | **0.5** | **0.6** | **0.7** | **0.8** | **0.9** | **1** |
| **OS HR** | **0** | 24,080 | 26,353 | 28,631 | 30,921 | 33,229 | 35,561 | 37,922 | 40,315 | 42,745 | 45,214 | 47,724 |
|  | **0.1** | 24,080 | 25,530 | 27,808 | 30,096 | 32,398 | 34,723 | 37,074 | 39,455 | 41,872 | 44,326 | 46,821 |
|  | **0.2** | 24,056 | 24,679 | 26,952 | 29,231 | 31,522 | 33,832 | 36,166 | 38,528 | 40,923 | 43,354 | 45,824 |
|  | **0.3** | 23,801 | 23,807 | 25,846 | 28,091 | 30,346 | 32,615 | 34,906 | 37,222 | 39,569 | 41,949 | 44,365 |
|  | **0.4** | 23,380 | 23,380 | 24,589 | 26,786 | 28,989 | 31,205 | 33,439 | 35,696 | 37,980 | 40,295 | 42,645 |
|  | **0.5** | 22,879 | 22,879 | 23,310 | 25,428 | 27,574 | 29,729 | 31,901 | 34,092 | 36,309 | 38,554 | 40,831 |
|  | **0.6** | 22,345 | 22,345 | 22,358 | 24,074 | 26,161 | 28,254 | 30,361 | 32,486 | 34,633 | 36,807 | 39,009 |
|  | **0.7** | 21,804 | 21,804 | 21,787 | 22,758 | 24,784 | 26,816 | 28,585 | 30,917 | 32,995 | 35,098 | 37,228 |
|  | **0.8** | 21,275 | 21,275 | 21,275 | 21,627 | 23,466 | 25,437 | 27,418 | 29,412 | 31,424 | 33,459 | 35,519 |
|  | **0.9** | 20,770 | 20,770 | 20,770 | 20,791 | 22,216 | 24,130 | 26,051 | 27,984 | 29,933 | 31,903 | 33,895 |
|  | **1** | 20,284 | 20,284 | 20,284 | 20,257 | 21,066 | 22,887 | 24,751 | 26,625 | 28,514 | 30,422 | 32,351 |

VBPs are calculated for combinations of hazard ratios (HRs) ranging from 0.1 to 1.0. One interesting insight from this analysis is the counterintuitive positive correlation between the PFS HR value and the VBP value (this is because the pre-progression costs in this model are considerably higher than the post-progression costs, so the QALY gain associated with a lower progression rate is offset by the associated increase in costs).

## Supplementary Material D: Cost-effectiveness Analysis Base Case Settings

| **Aspects of Model** | **Key Assumptions** | **Justification** |
| --- | --- | --- |
| Model time horizon | 10 years | The model assumes a 10-year time horizon, which is assumed to correspond to a lifetime horizon since only a small number of patients survive beyond that period. |
| Discount rate for costs and QALYs | 3.5% | In line with the NICE reference case |
| Patient age | 72.0 (range: 38–96) years | Rischin D, et al 2021 |
| Proportion of male patients | 83.4% | Rischin D, et al 2021 |
| Long-term remission | Not included in the base case | Explored in the scenario analysis |
| PFS HR: Dummymab vs. Cemiplimab | 0.40 [95% CI : 0.30, 0.50] | Assumption |
| OS HR: Dummymab vs. Cemiplimab | 0.50 [95% CI : 0.40, 0.60] | Assumption |
| Survival distribution (PFS) | Log-normal | Assumption for illustration |
| Survival distribution (OS) | Log-normal | Assumption for illustration |
| Pre-progression utility | 0.793 | NICE TA592 |
| Post-progression utility | 0.701 | NICE TA592 |
| Age-related adjustment on utility | Yes, utilities were adjusted by UK general population utility where utility decreases with age | Based on the Ara and Brazier study suggesting the impact of age on HRQoL. |
| Incidence of AEs | Grade ¾ adverse events occurring in ≥1% of patients. All AEs were assumed to last 30 days | Assumption based on NICE TA592 |
| List price of Dummymab (intervention) | £1723 per vial (£29,963 per year) | Assumption |
| List price of cemiplimab (comparator) | £4,650 per 350 mg vial | NICE TA592 |
| PAS price discount applied on cemiplimab | 60% per 350mg vial | Assumption |

## Supplementary Material E: One-way Sensitivity Analysis

A one-way sensitivity analysis was conducted to show the extent to which the VBP was impacted with selected parameter values were varied. See Supplementary Material B: Illustration of model structure and details of model parameters for details on the upper and lower bounds used in the one-way sensitivity analysis.

Figure 5: OWSA for dummymab VBP (GBP). Top 10 parameters are included. Note that higher PFS utility values do not alter the VBP as there is an age-related upperbound on utility values used in the model

## Supplementary Material F: Estimation of value drivers for *dummymab* using ‘switch-on’ method

The two differentiators between dummymab and cemiplimab were envisioned, as per the TPP, to be improvement in PFS (HR=0.4) and improvement in OS (HR=0.5). These differentiators can be switched on in the cost-effectiveness model to show how the total VBP is reached, as per the figures below. These two figures differ only by the order in which the clinical differentiators were ‘swtiched on’, demonstrating the inherent unreliability of the method. These figures also give further evidence to the counterintuitive feature in this analysis of an improvement in PFS leading to a disimprovement in cost-effectiveness.

Estimate of value drivers (GBP) using the switch-on method where the OS HR is switched on first

Estimate of value drivers (GBP) using the switch-on method where the PFS HR is switched on first

## Supplementary Material G: Comparative Summary of Healthcare System VoI with Manufacturer VoI

| **Aspect** | **Healthcare System VOI** | **Manufacturer VOI** |
| --- | --- | --- |
| **Decision uncertainty** | Pr(technology is cost-effective) | Pr(Target Price ≤ Value-based Price) |
| **Payoff with adoption** | Net health benefit to population | Revenue from sales |
| **Payoff without adoption** | Current care net benefit | Zero revenue |
| **Key assumption** | Adopt if cost-effective | Launch if expected cost-effective |
